# Autism cornered: network analyses reveal mechanisms of autism spectrum disorders

**DOI:** 10.15252/msb.20145937

**Published:** 2014-12-30

**Authors:** Charles Auffray

**Affiliations:** European Institute for Systems Biology & Medicine, CNRS-ENS-UCBL, Université de LyonLyon, France

## Abstract

Despite a wealth of behavioral, cognitive, biological, and genetic studies, the causes of autism have remained largely unknown. In their recent work*,* Snyder and colleagues (Li *et al*, 2014) use a systems biology approach and shed light on the molecular and cellular mechanisms underlying autism, thus opening novel avenues for understanding the disease and developing potential treatments.

See also: **J Li *et al*** (December 2014)

A variety of conditions that perturb children in their early social development and impair their ability to communicate with others have been grouped as autism spectrum disorders (ASD) to reflect their wide range of common symptoms. The best known in the general public are repetitive behaviors and cognitive changes that can lead to intellectual disability or on the contrary to unusual skills such as systematic memorizing of long lists of events or instant numbering of elements as demonstrated by Dustin Hoffman alias Charlie Babbitt in the movie Rain Man a quarter century ago.

Autism in its different forms has increased significantly in recent years, becoming a health problem of growing social concern as it affects young children and their families. The pervasive but elusive nature of ASD is reflected in more than 5,000 publications registered in PubMed, which highlight the complex interplay of the numerous environmental, biochemical, and genetic risk factors involved. Accordingly, the diagnosis and treatment of ASD has been focusing on empirical attempts to manage specific symptoms using a combination of behavioral, nutritional, or pharmacological interventions (Compart, 2013).

In spite of evidence from twin and family studies for an inherited component in the risk to develop ASD, genomewide associations studies (GWAS) focusing on common polymorphisms have failed to identify replicable risk loci, which are usually associated with very small changes between ASD cases and controls. The situation has changed with the shift in focus on rare variants and copy number variations with large effects, resulting in the identification of several hundred risk loci (Willsey & State, 2015), and recent work has started to link them to the pathophysiology of ASD through the integration of multiple complex datasets into biological pathways, networks, and circuits (Parikshak *et al*, 2013; Hormozdiari *et al*, 2014).

This follows a trend that was initiated in the field of cancer over the past decade (Chuang *et al*, 2007) and is now being implemented in other biomedical fields such as respiratory medicine (Turan *et al*, 2011; Gustafsson *et al*, 2014). These approaches are predicated on the assumption that the use of prior knowledge enables overcoming the limitations encountered in GWAS (Ideker *et al*, 2011). However, metrics for assessing the significance of findings obtained by combining heterogeneous datasets remain to be developed, thus increasing the need for stringent experimental challenge and validation of the generated hypotheses.

This is the route taken by Snyder and colleagues (Li *et al*, 2014) in a bold attempt to leverage a number of rich sources of data and knowledge and to complement them with relevant additional measurements to unravel the molecular networks of ASD (Fig1). They first curated the human protein interactome through co-expression analysis and by topological decomposition they identified over 800 modular components. These modules were examined for the presence of the 383 ASD-related genes reported in the literature, thus identifying a number of modules highly enriched in ASD-related genes, each of which represents a working hypothesis worth further exploration. They then focused their attention on a module of 119 genes (module #13) enriched in ASD-related genes, and by comparing whole genome or exome sequences of 25 ASD patients to those of 4 control subjects, they detected 113 nonsynonymous mutations in 38 genes in this module, 28 of which are associated with ASD for the first time.

**Figure 1:**
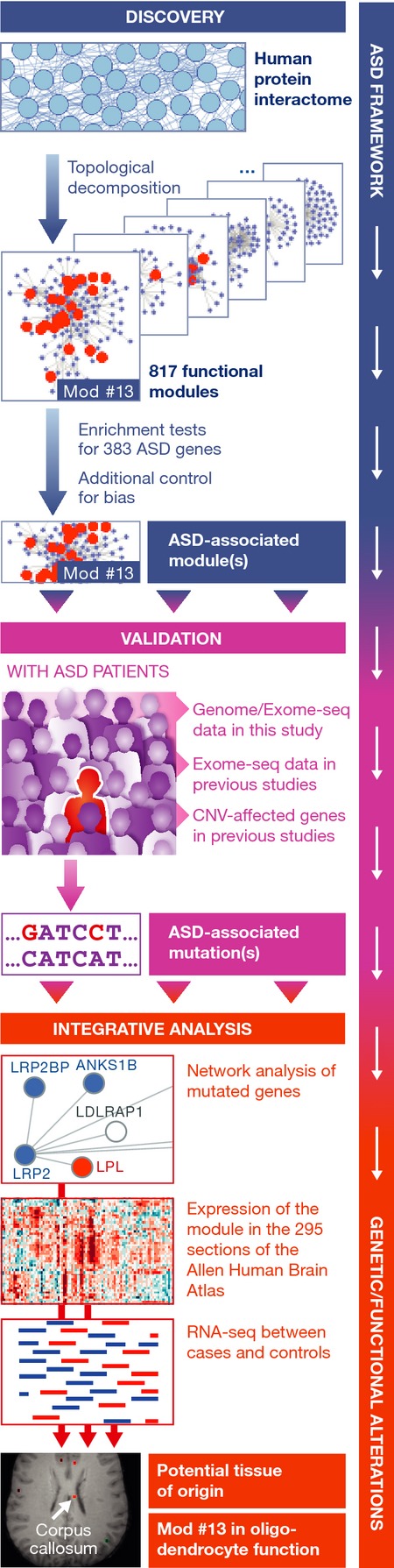
Network analyses reveal a functional module linked to autism The human protein interactome was modularized by topological decomposition. ASD-associated modules were identified based on the presence of ASD-related genes. Validation analyses using newly generated and existing exome/genome sequencing data indicated enrichment for rare nonsynonymous mutations in the ASD-related module #13. Functional characterization of the module by network and transcriptome analyses indicated the corpus callosum as a potential tissue of origin of ASD and provided evidence for a function of the module in oligodendrocyte maturation (MRI image of the corpus callosum: Allen Institute of Brain Science).

These findings were confirmed through the re-analysis of the exomes sequenced in a study of a large independent cohort of ASD and controls, which had failed to detect them. The relevance of the newly identified ASD-related genes was then assessed through analysis of their expression patterns in the Allen Human Brain Atlas, revealing a subset of genes in the module expressed specifically in the corpus callosum, a large white matter structure rich in oligodendrocytes interfacing the brain hemispheres for communication of motor, sensory, and cognitive signals, known to be reduced in size in ASD patients. Analysis of the expression of the mouse orthologs provided supporting evidence that the subset of genes is functionally associated with oligodendrocyte maturation. Closing the loop, the authors established, through deep RNA sequencing of ASD samples, that the expression of these genes is altered in the corpus callosum. Altogether, the accumulation in ASD patients of pathogenic mutations in genes that have a central position in the interactome network lends credence to the hypothesis that ASD phenotypes result from a disruption of interhemispheric communication related to changes in interconnected genes that trigger a decrease of oligodendrocyte maturation.

Undoubtedly, much work remains to be done to consolidate these findings into diagnostic and therapeutic tools, and the paper provides a large knowledge base and set of modules as working hypotheses worth exploring in relation to ASD. It is worth emphasizing that this and similar studies using systems approaches would not be possible without the availability of openly shared high-quality datasets, which can be interrogated in novel ways by other investigators and complemented by further relevant information ranging from high-resolution imaging to high-throughput and time-resolved functional and omics data. The study by Li *et al* is exemplar in this respect, as it contributes to the development of an openly shared methodological framework and tools for data analysis and integration that can be used to explore the complexity underlying many other rare or common diseases.
